# A pan-cancer analysis of FAT atypical cadherin 4 (FAT4) in human tumors

**DOI:** 10.3389/fpubh.2022.969070

**Published:** 2022-08-16

**Authors:** Weili Mao, Jiajing Zhou, Jie Hu, Kui Zhao, Zhenling Fu, Jun Wang, Kaili Mao

**Affiliations:** ^1^Department of Pharmacy, The Quzhou Affiliated Hospital of Wenzhou Medical University, Quzhou People's Hospital, Quzhou, China; ^2^Department of Oncology, Yantai Hospital of Traditional Chinese Medicine, Yantai, China; ^3^School of Integrative Medicine, Tianjin University of Traditional Chinese Medicine, Tianjin, China

**Keywords:** pan-cancer analysis, FAT4, prognosis, clinical stages, immune infiltration

## Abstract

**Objective:**

We performed a pan-cancer analysis to explore the potential mechanisms of FAT4 in 33 different tumors.

**Methods:**

In this study, we selected 33 types of cancers based on the datasets of TCGA (the cancer genome atlas). We analyzed the expression of FAT4 in tumor and normal tissues. Meanwhile, we analyzed the expression levels of FAT4 in tissues from tumors of different stages. Kaplan-Meier survival analysis, Tumor Mutational Burden (TMB), Microsatellite Instability (MSI), immune infiltration analysis, Gene set enrichment analysis (GSEA), and FAT4-related gene enrichment analysis were performed.

**Results:**

FAT4 expression in most tumor tissues was lower than in corresponding control tissues. FAT4 expression was different in different stages of bladder cancer (BLCA), kidney clear cell carcinoma (KIRC), and breast cancer (BRCA). In addition, the expression level of FAT4 in different types of tumors has an important impact on the prognosis of patients. FAT4 might influence the efficacy of immunotherapy via tumor burden and microsatellite instability. We observed a statistically positive correlation between cancer-associated fibroblasts and FAT4 expression in most tumors. GSEA of BLCA indicated that low FAT4 expression groups were mainly enriched in calcium signaling pathway and chemokine signaling pathway. GSEA analysis of KIRC suggested low FAT4 expression groups were mainly involved in olfactory transduction and oxidative phosphorylation. Kyoto Encyclopedia of Genes and Genomes (KEGG) indicated that the role of FAT4 in the pathogenesis of cancer may be related to human papillomavirus infection, Hippo signaling pathway, PI3K–Akt signaling pathway, etc. Gene Ontology (GO) enrichment analysis further showed that most of these genes were related to the pathways or cell biology, such as peptidyl–tyrosine phosphorylation, cell junction assembly, protein tyrosine kinase activity, etc.

**Conclusion:**

Our study summarized and analyzed the antitumor effect of FAT4 in different tumors comprehensively, which aided in understanding the role of FAT4 in tumorigenesis from the perspective of clinical tumor samples. Pan-cancer analysis showed that FAT4 to be novel biomarkers for various cancers prognosis.

## Introduction

Due to the complexity of tumorigenesis, in recent research, more attention has been placed on the pan-cancer analysis of various genes of interest, which not only contributes to clinical oncology practice, but also helps us understand clinical prognosis and underlying molecular mechanisms. Human FAT family (FAT−4) proteins are human homologs of Drosophila fat and are involved in cell growth, tumor suppression and planar cell polarity (1). More and more evidences show that the downregulation of FAT4 may be related to the pathogenesis of several malignancies, and FAT4 has been identified as a tumor suppressor in various cancers, including gastric, breast and colorectal cancer (2–4), which contributes to the inhibition of cell proliferation and invasiveness, and thus suppresses metastasis. In particular, FAT4 has been considered as a new biomarker for breast cancer prognosis (5). The Cancer Genome Atlas (TCGA) is a publicly funded project that aims to catalog and discover major cancer-causing genomic alterations to create a comprehensive “atlas” of cancer genomic profiles, so far, TCGA researchers have analyzed large cohorts of 37 types of genomic and clinical data for 33 cancer types through large-scale genome sequencing and integrated multi-dimensional analyses (6).

In this study, we comprehensively detected FAT4 in 33 different tumors and analyzed the FAT4 expression and correlation between FAT4 expression and prognosis of cancer patients to clarify the potential molecular mechanism of FAT4 in tumorigenesis through data from the TCGA project. This study contributes to further understanding of the abnormal expression of FAT4 and provides a theoretical basis for for clinical targeted therapy of FAT4.

## Methods

### Data sources

UCSC Xena was developed as a high-performance visualization and analysis tool for both large public repositories and private datasets. Xena's privacy-aware architecture enables cancer researchers of all computational backgrounds to explore large, diverse datasets from TCGA, Pan-Cancer Atlas, PCAWG, ICGC, GTEx, and the GDC (7, 8).

We accessed TCGA data through UCSC Xena website (https://xena.ucsc.edu/) to obtain Gene Expression RNA sequencing (RNA seq) data, Somatic Point Mutation (SPM) data, phenotype data and matched clinical data (*n* = 12,591). Pan-Cancer Atlas Hub of 33 different cancer types (as shown in Table 1).

**Table 1:** 33 different cancer types and its abbreviation.

**Cancer type**	**Abbreviation**
Acute myeloid leukemia	LAML
Adrenocortical cancer	ACC
Bile duct cancer	CHOL
Bladder cancer	BLCA
Breast cancer	BRCA
Cervical cancer	CESC
Colon cancer	COAD
Endometrioid cancer	UCEC
Esophageal cancer	ESCA
Glioblastoma	GBM
Head and neck cancer	HNSC
Kidney chromophobe	KICH
Kidney clear cell carcinoma	KIRC
Kidney papillary cell carcinoma	KIRP
Large B-cell lymphoma	DLBC
Liver cancer	LIHC
Lower grade glioma	LGG
Lung adenocarcinoma	LUAD
Lung squamous cell carcinoma	LUSC
Melanoma	SKCM
Mesothelioma	MESO
Ocular melanomas	UVM
Ovarian cancer	OV
Pancreatic cancer	PAAD
Pheochromocytoma & paraganglioma	PCPG
Prostate cancer	PRAD
Rectal cancer	READ
Sarcoma	SARC
Stomach cancer	STAD
Testicular cancer	TGCT
Thymoma	THYM
Thyroid cancer	THCA
Uterine carcinosarcoma	UCS

### Gene expression analysis

By using Gene Expression RNA seq data from the TCGA project, we compared the expression differences of FAT4 in TCGA tumor tissues and normal tissues, and evaluated the potential of FAT4 as a therapeutic target for treating tumors. Through R software and its program packages “Limma”, “Plyr”, “Reshape2”, and “Ggpubr”, we obtained the violin plots of FAT4 expression difference between tumor tissue and normal tissue. A value of *P*-value < 0.05 indicates a significant difference between tumor tissue and normal tissue. The smaller the *P*-value, the more significant the difference.

### Survival analysis

Clinical status and outcome are important indicators of clinical status. We used Kaplan-Meier survival analysis to evaluate the relationship between FAT4 and prognosis of tumor patients, calculate its correlation and draw the survival curve of the prognosis of patients, including overall survival (OS), disease specific survival (DSS), disease free survival (DFS), progression free survival (PFS) and expression levels of FAT4 in 33 different cancer types. Log-rank test and Cox regression analysis were used to perform survival analysis of high and low expression groups, and hazard ratios (HR) were determined with 95% confidence intervals and *p* values. A *P*-value < 0.05 was considered statistically significant.

### Correlation analysis of FAT4 expression with TMB and MSI

As a quantifiable biomarker, tumor mutational burden (TMB) can be used to reflect the number of mutations contained in tumor cells (9). Microsatellite Instability (MSI) has been considered for use as the potential biomarker to predict the efficacy of tumor immunotherapy (10). The mutation data of 33 different tumors were acquired from the TCGA database, and the TMB and MSI were calculated by R software and its “fmsb” packages.

### Immune infiltration analysis

To analyze the role of immune cells in the tumor microenvironment, we used the “Immune-Gene” module of TIMER2.0 (http://timer.cistrome.org/) to analyze the expression profile data of all tumor tissues in TCGA. The TIMER, CIBERSORT, CIBERSORT-ABS, QUANTISEQ, XCELL, MCPCOUNTER, and EPIC algorithms were used for immune infiltration analysis, and cancer-associated fibroblasts were selected. The *P*-values and partial correlation (cor) values were derived by using purity-adjusted Spearman's rank correlation test. Data were visualized using the heatmap and scatter plot.

### FAT4-related gene enrichment analysis

The STRING (https://cn.string-db.org/) was used to obtain the available experimentally determined FAT4-binding proteins (FAT4; homo sapiens) with the settings of minimum required interaction score: low confidence 0.150; network type: full network; maximum number of interactions shown: no more than 50 interactors; meaning of network edge: evidence. We used the “Similar Gene Detection” module of GEPIA2 (http://gepia2.cancer-pku.cn/#index) to screen the top 100 FAT4-related targeted genes. We used the “correlation analysis” module of GEPIA2 to analyze the correlation between FAT4 and top 5 genes with Pearson correlation, and the results were presented as dot plots. Thereafter, we also used the “Gene_Corr” module of TIMER2.0 (http://timer.cistrome.org/) to obtain the expression of the top 5 target genes in each tumor and displayed them in the form of heatmaps. We merged FAT4-binding proteins and FAT4-related targeted genes. Then KEGG pathway analysis and GO enrichment analysis were performed on the combined results by R software. Furthermore, Gene set enrichment analysis (GSEA) was performed to investigate biological signaling pathways of statistically significantly cancers by R software and its packages “limma”, “org.Hs.eg.db”, “clusterProfiler”, etc.

## Results

### Gene expression analysis

**Figure 1:**
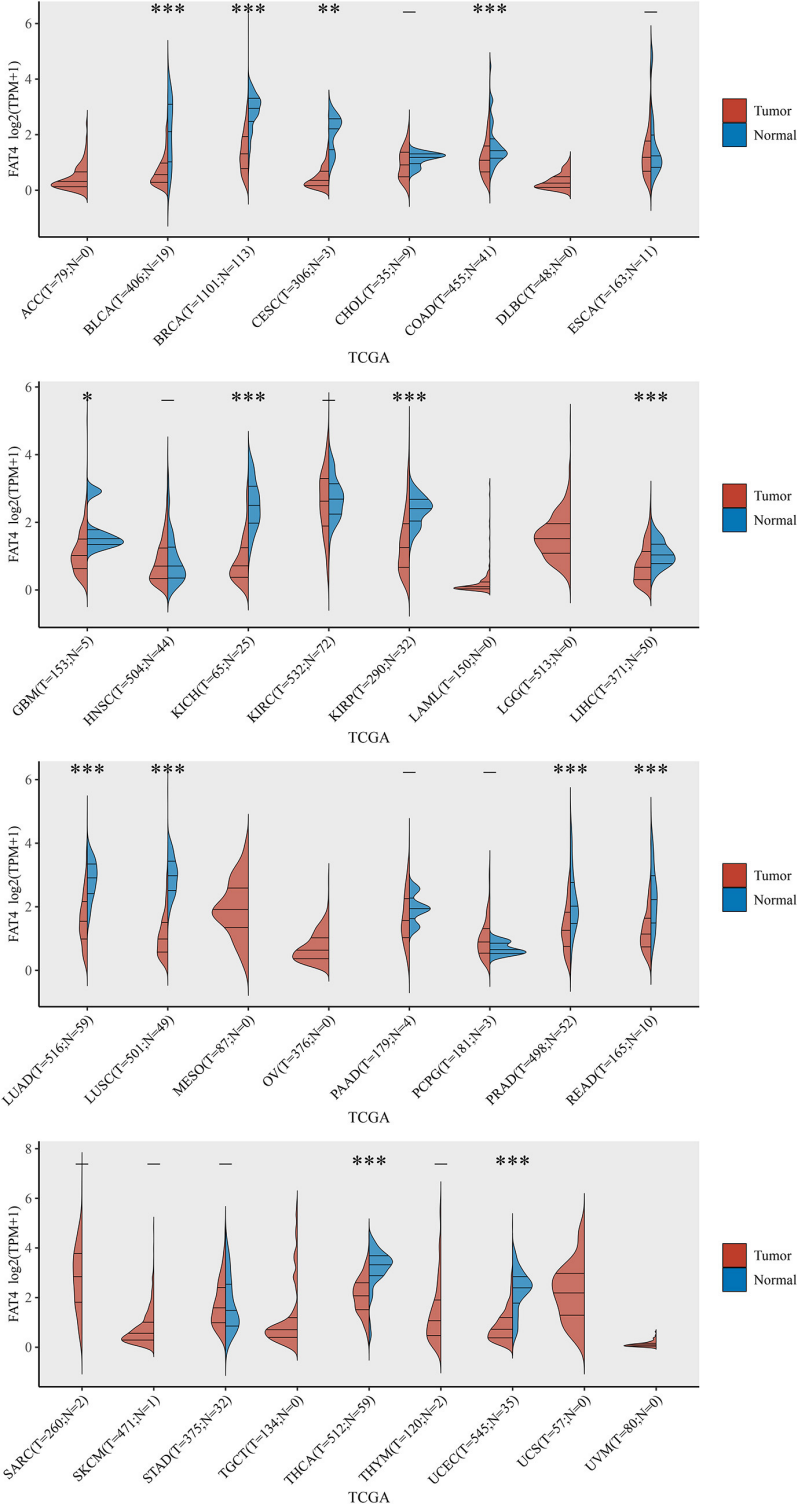
The expression level of FAT4 gene in different tissues. Red represents the tumor tissue, blue represents the normal tissue, X-axis represents the tumor type, and Y-axis represents the expression level of FAT4. *Appears above the tumor, FAT4 expression level is different between the tumor group and the normal group in this tumor type. *Represents *P*-value < 0.05; **represents *P*-value < 0.01; ***represents *P*-value < 0.001.

We found that FAT4 expression in tumor tissues of BLCA, BRCA, COAD, KICH, KIRP, LIHC, LUAD, LUSC, PRAD, READ, THCA, UCEC *(P* < 0.001), CESC(*P* < 0.01), and GBM (*P* < 0.05) was lower than that in the control tissues corresponding to the TCGA dataset, but we did not find significant differences in other tumors, such as ACC, DLBC, and ESCA (Figure 1).

### FAT4 clinical correlation analysis

**Figure 2:**
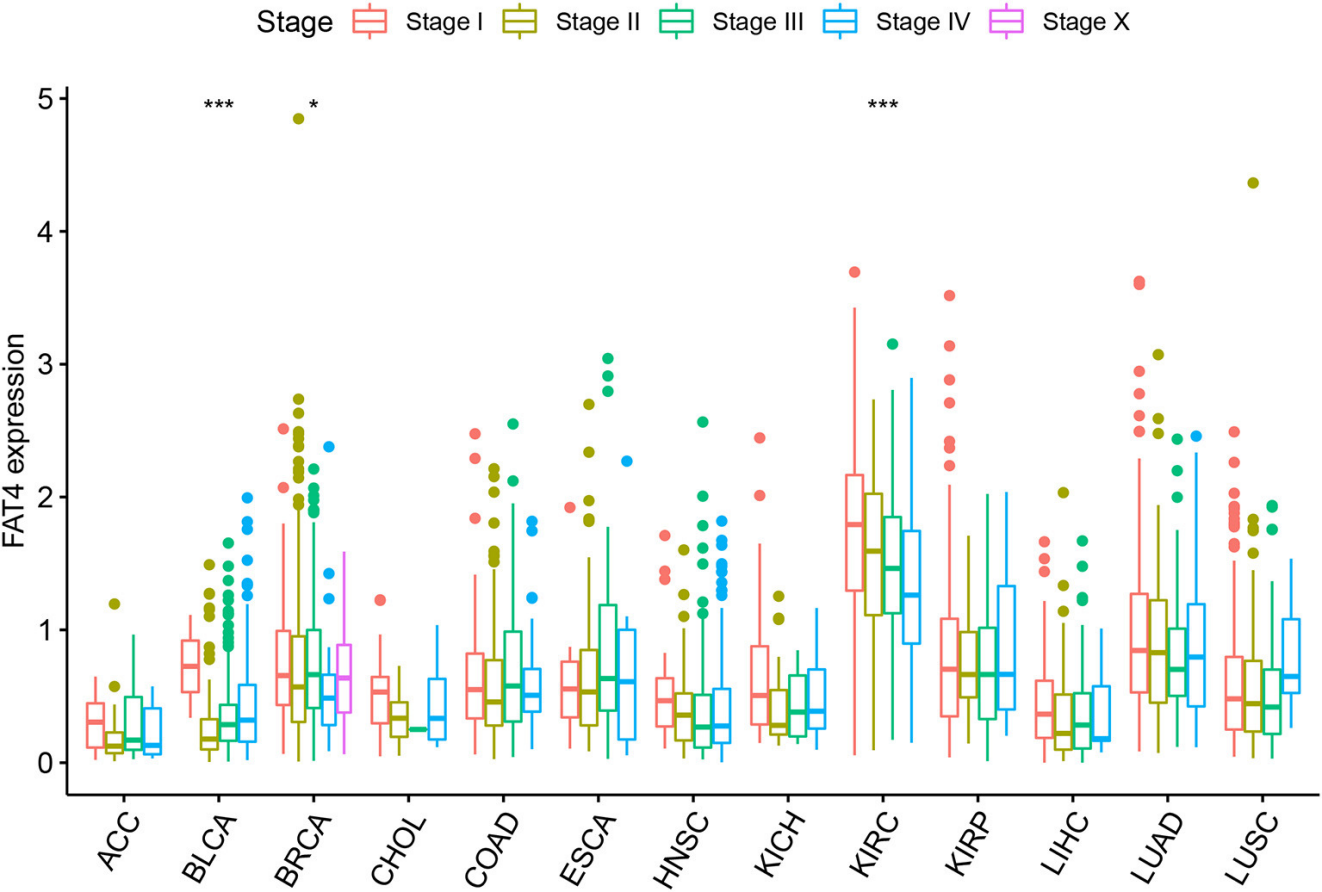
Relationship between FAT4 and clinical stage. X-axis represents the tumor type, and Y-axis represents the expression level of FAT4. *Appears above the tumor, FAT4 expression levels is different in different stages of the tumor group in this tumor type; *represents *P*-value < 0.05; ***represents *P*-value < 0.001.

By analyzing the expression levels of FAT4 in tumor tissues at different stages, we found that FAT4 expression was different in different stages of BLCA, KIRC, and BRCA. In other tumors, there was no significant difference in tumor stage and expression level (Figure 2). FAT4 expression was low in KIRC tissues, and the expression of Stage II, Stage III, and Stage IV was lower than that of Stage I. In BRCA tissues, the expression level of Stage IV was the lowest, and the expression levels of Stage I and Stage III were relatively higher. In BLCA tissues, the expression level of Stage II was the lowest, and the expression level of Stage I was relatively higher.

### Survival analysis

**Figure 3:**
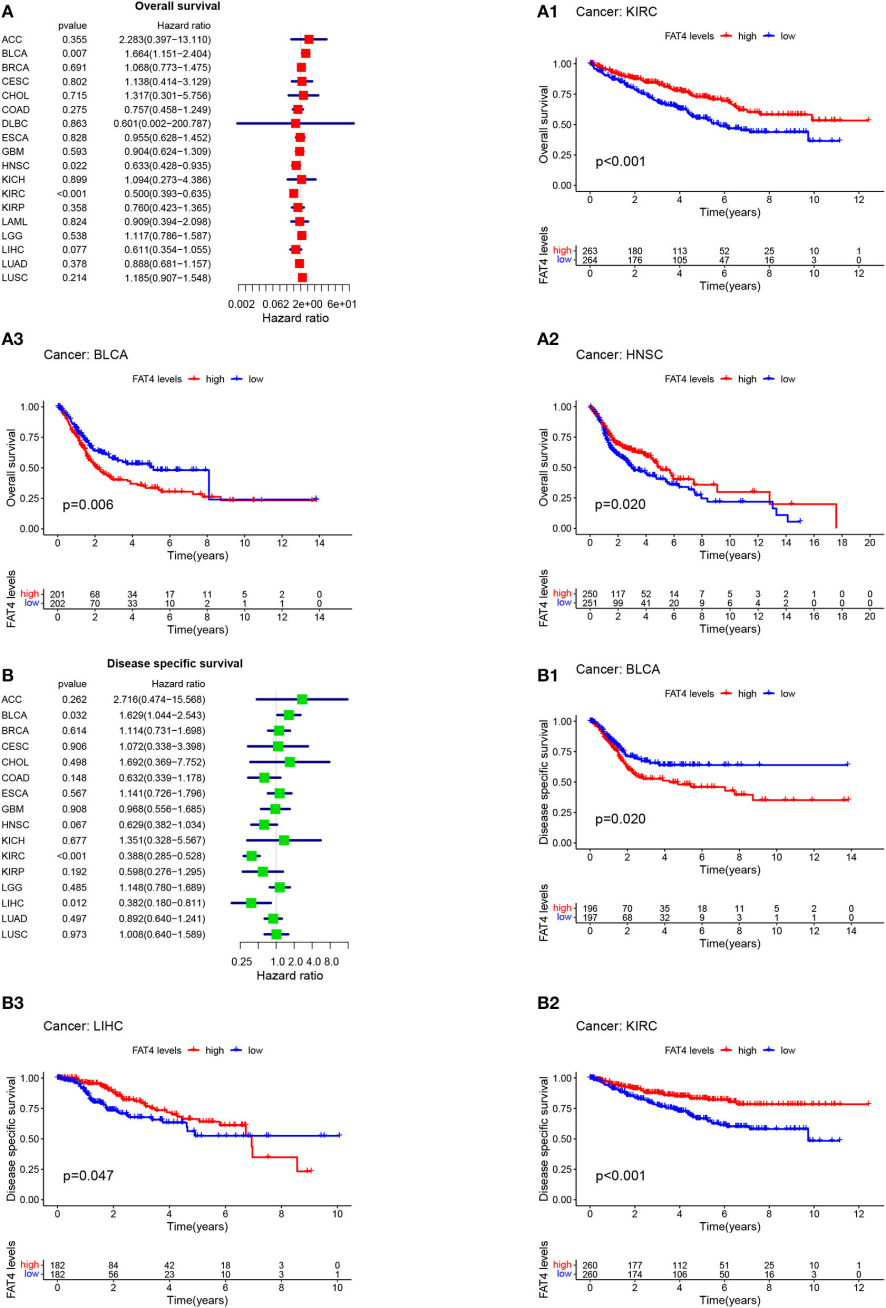
Correlation between FAT4 expression and survival prognosis of cancers. **(A,B)** Forest plots of OS and DSS. **(A1–B3)** Kaplan-Meier curves for OS and DSS.

**Figure 4:**
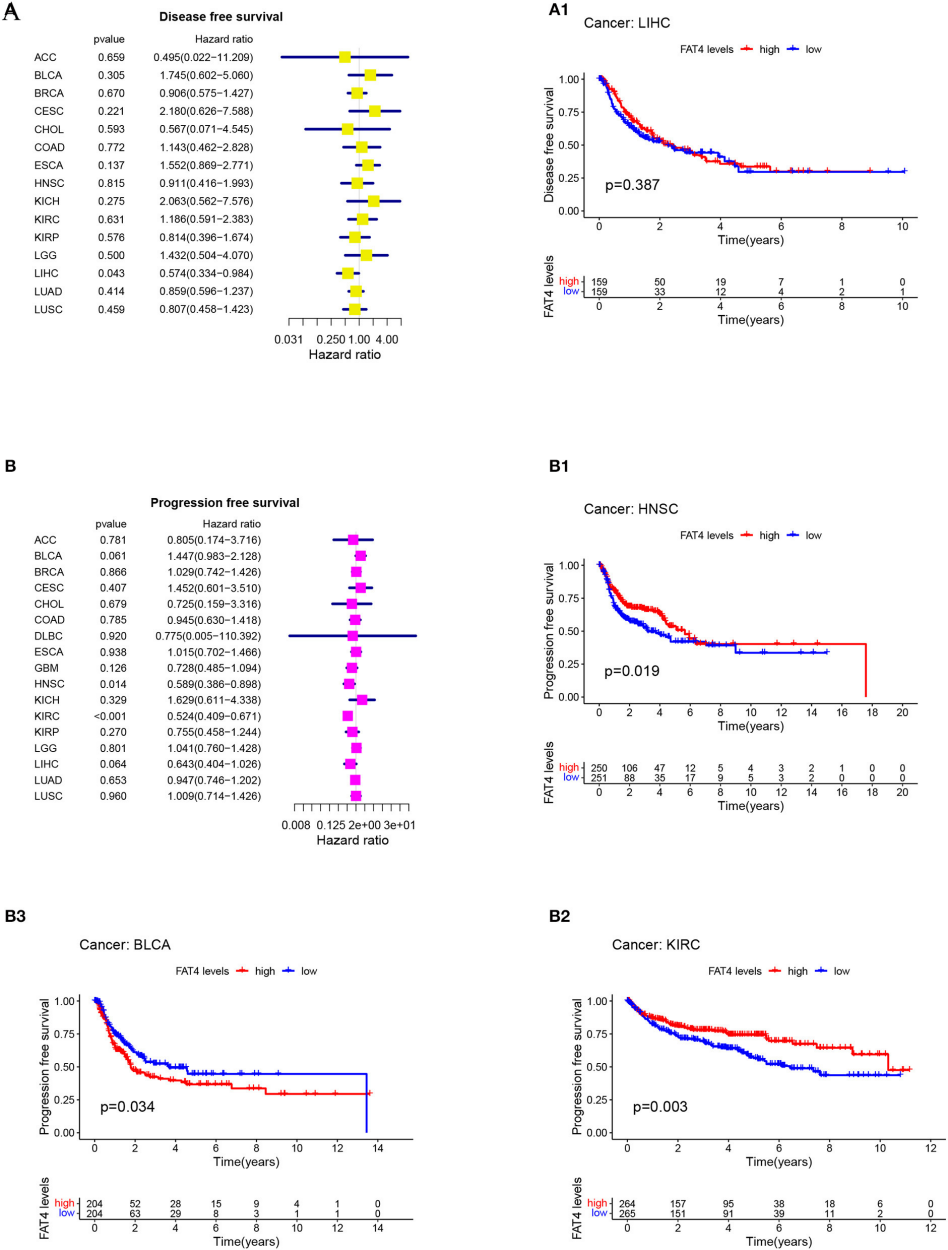
Correlation between FAT4 expression and survival prognosis of cancers. **(A,B)** Forest plots of DFS and PFS. **(A1–B3)** Kaplan-Meier curves for DFS and PFS.

Patient prognosis is an important clinical evaluation index. For survival analysis, the Kaplan-Meier method was used to analyze the correlation between FAT4 and tumor prognosis. The results of survival analysis indicated that increased FAT4 expression levels negatively correlated with OS, DSS, and PFS significantly of BLCA patients while positively correlated with OS of HNSC patients and KIRC patients; with DSS of LIHC patients and KIRC patients (Figure 3); with DFS of LIHC patients; with PFS of HNSC patients and KIRC patients (Figure 4), that is, FAT4 is a high-risk gene for OS, DSS, and PFS of BLCA patients. The higher the expression of FAT4, the greater the risk and the worse the prognosis of patients. FAT4 is a low-risk gene for OS and PFS in HNSC patients, for OS, DSS, and PFS of KIRC patients, and for DSS and DPS of LIHC patients. The higher the expression of FAT4, the lower the risk and the better the prognosis of patients.

### Correlation analysis with TMB and MSI

**Figure 5:**
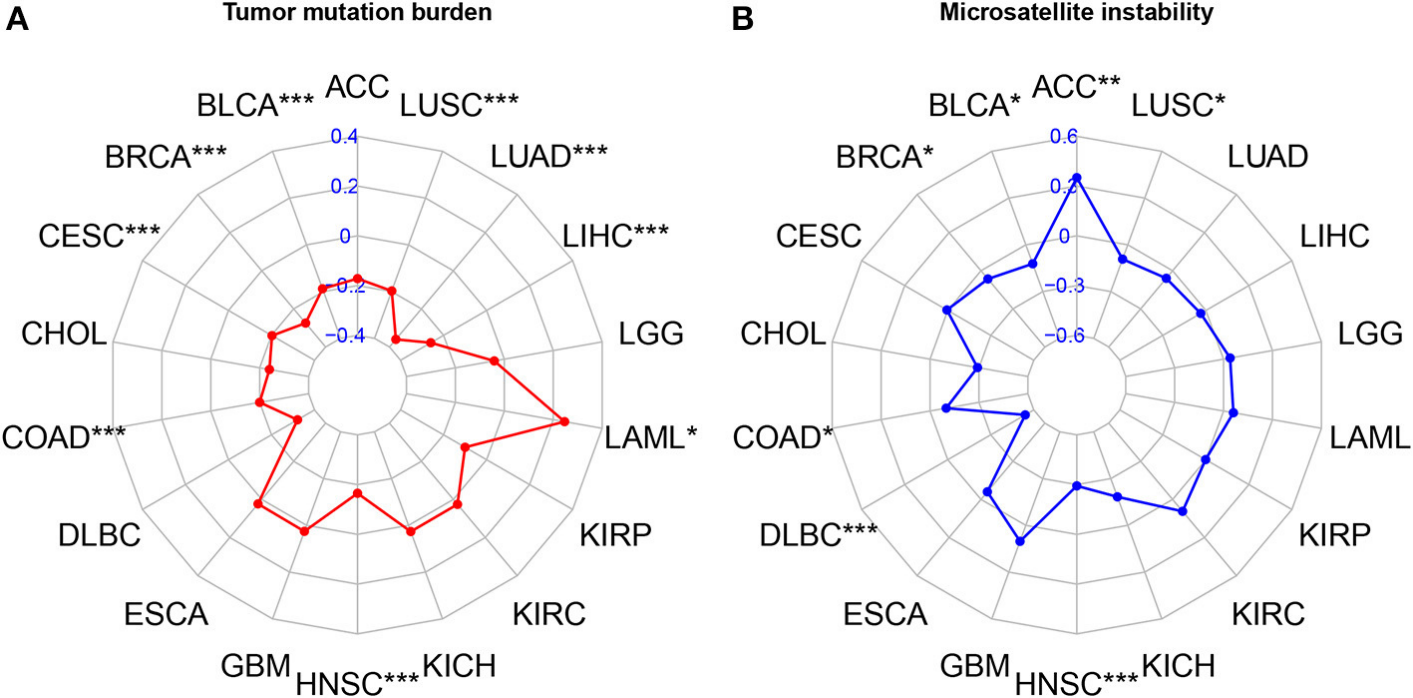
Correlation analysis with TMB and MSI. **(A)** Correlation analysis of FAT4 expression and Tumor mutation burden. **(B)** Correlation analysis of FAT4 expression and Microsatellite instability. *P < 0.05, **P < 0.01, ***P < 0.001.

The results of correlation analysis of FAT4 expression showed a significant positive correlation with TMB in THYM, PRAD (*P* < 0.001), KIRC, and OV (*P* < 0.05), while negatively correlated in LIHC, BRCA (*P* < 0.001), STAD, HNSC (*P* < 0.01), CESC, SKCM, ESCA, LUSC, PCPG, LAML, and PAAD (*P* < 0.05), however, there was no correlation were found in other tumors (Figure 5A). In addition, The results of correlation analysis of FAT4 expression showed a significant positive correlation with MSI in ACC (*P* < 0.001), while negatively correlated in HNSC, DLBC (*P* < 0.001), BLCA, BRCA, COAD, and LUSC (*P* < 0.05). There was no correlation were found in other tumors (Figure 5B).

### Immune infiltration analysis

**Figure 6:**
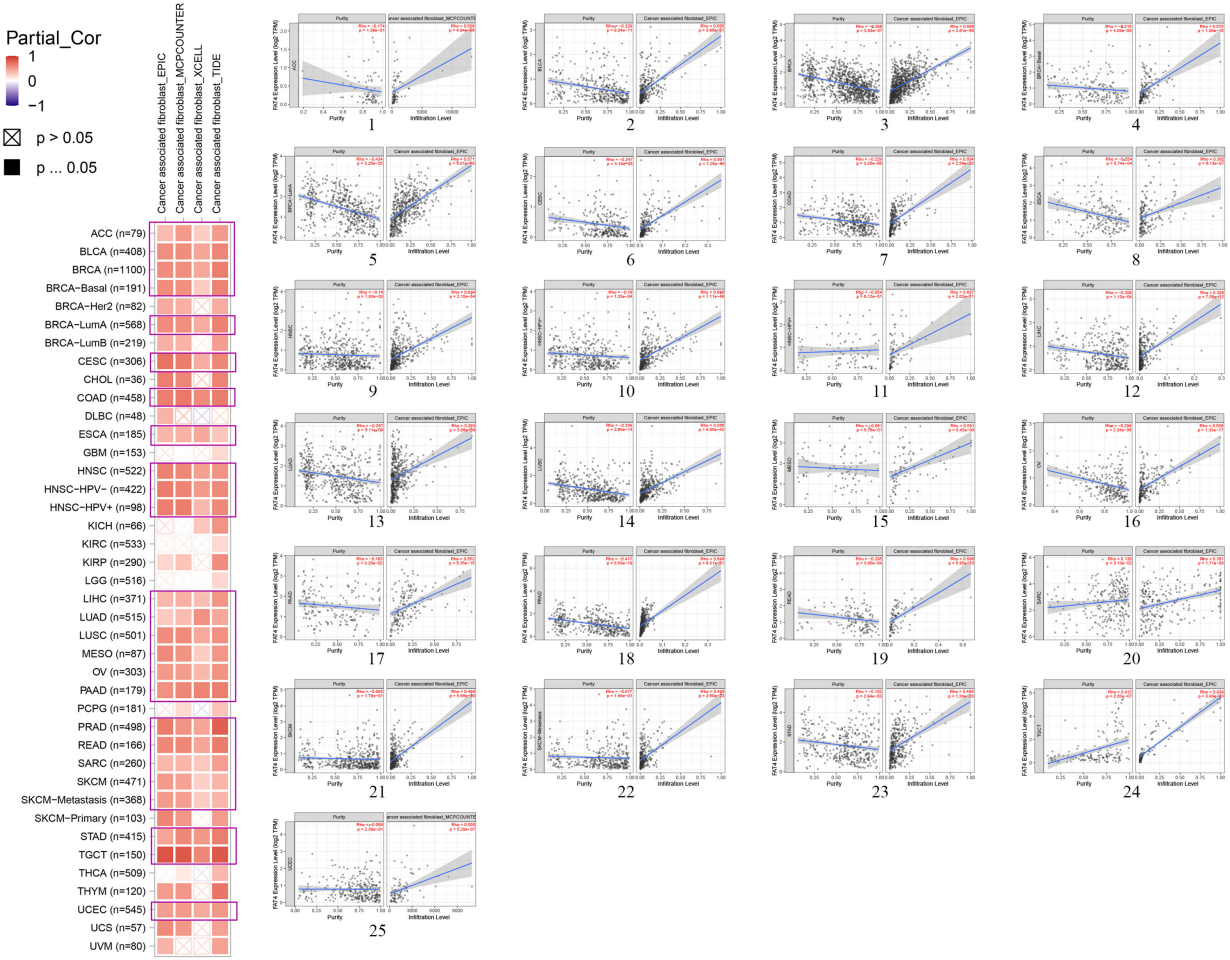
Correlation analysis between FAT4 expression and immune infiltration of cancer-associated fibroblasts. (1-25) Scatter plots of specific immune infiltration of cancer.

Our study adopted Estimate the Proportion of Immune and Cancer Cells (EPIC), marker genes expressions–based microenvironment cell populations-counter (MCPCOUNTER), gene signature enrichment–based xCell algorithm, Tumor Immune Dysfunction and Exclusion (TIDE) algorithms to evaluate results. After a sequence of analysis, we observed a statistically positive correlation between cancer-associated fibroblasts and FAT4 expression in the TCGA tumors of ACC, BLCA, BRCA (BRCA–Basal, BRCA–LumA), CESC, COAD, ESCA, HNSC (HNSC–HPV–, HNSC–HPV+), LIHC, LUAD, LUSC, MESO, OV, PAAD, PRAD, READ, SARC, SKCM (SKCM–Metastasis), STAD, TGCT, and UCEC (Figure 6).

### FAT4-related gene enrichment analysis

**Figure 7:**
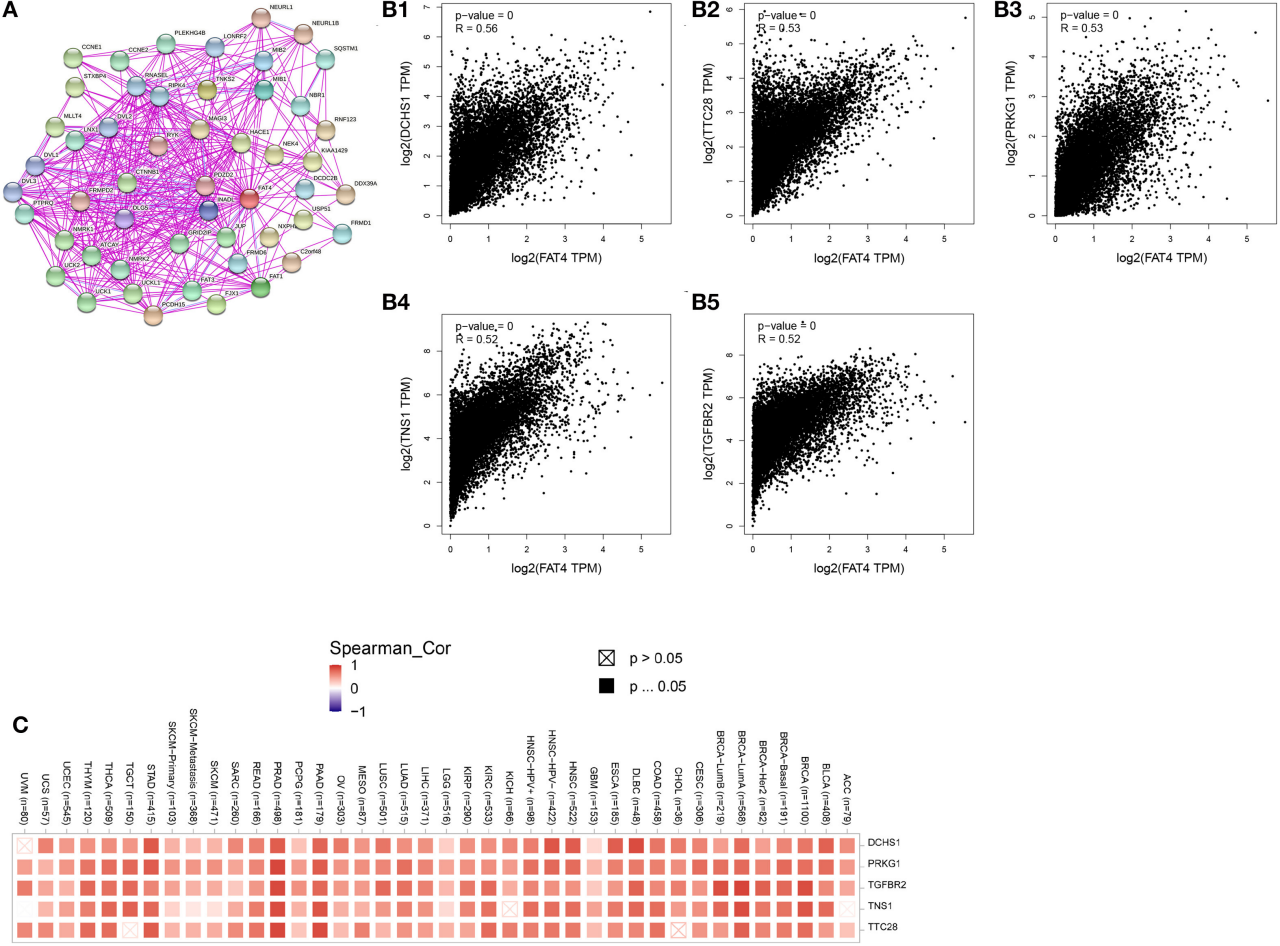
FAT4-Related Gene Analysis. **(A)** FAT4-binding proteins. **(B)** The correlation between FAT4 and top 5 FAT4-related targeted genes. **(C)** The expression of top 5 FAT4-related targeted genes in cancer.

**Figure 8:**
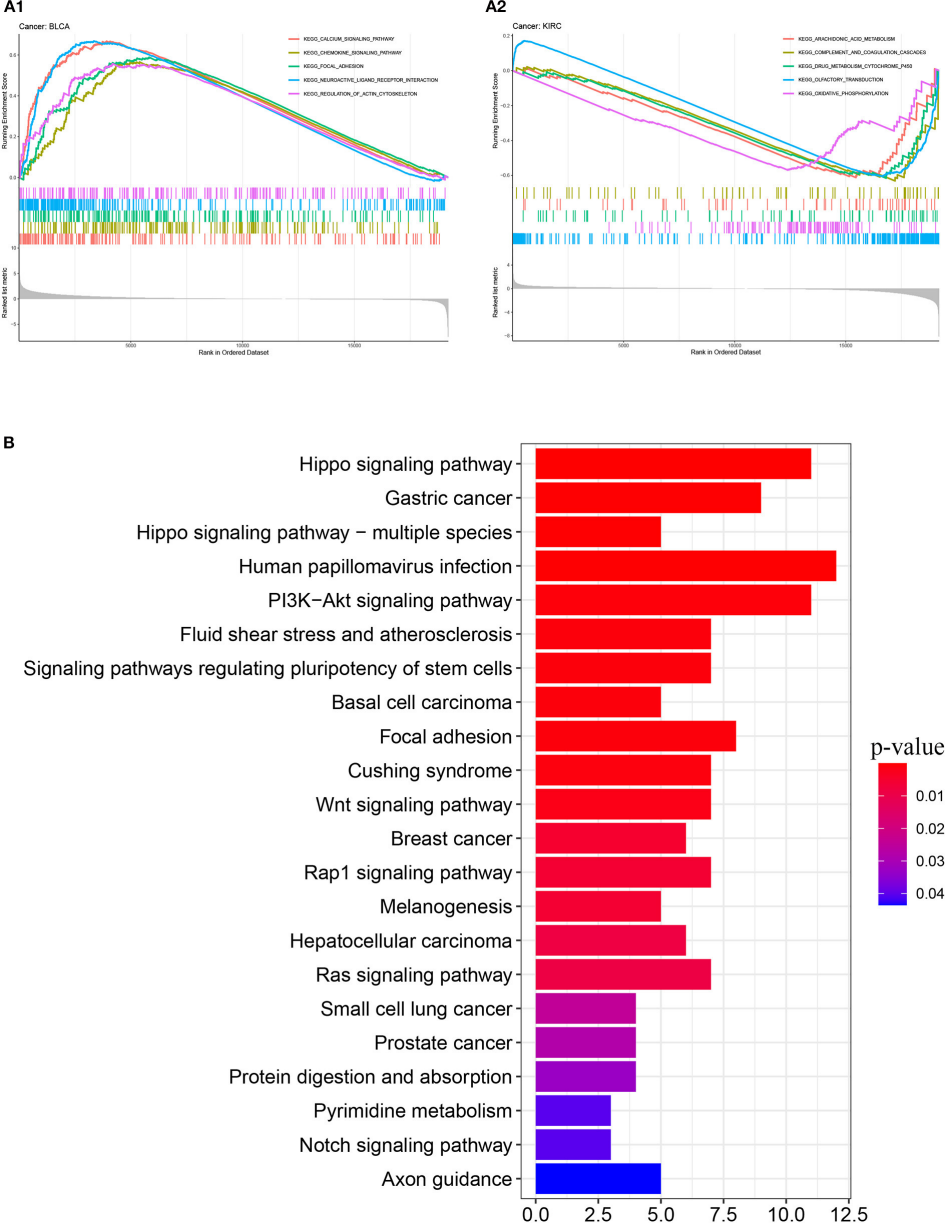
GSEA enrichment analysis and KEGG pathway analysis. **(A)** GSEA enrichment analysis with BLCA and KIRC. **(B)** KEGG pathway analysis. The y-axis shows top 20 significantly enriched pathway, and the x-axis displays the enrichment scores of these terms (*P*-value < 0.05).

**Figure 9:**
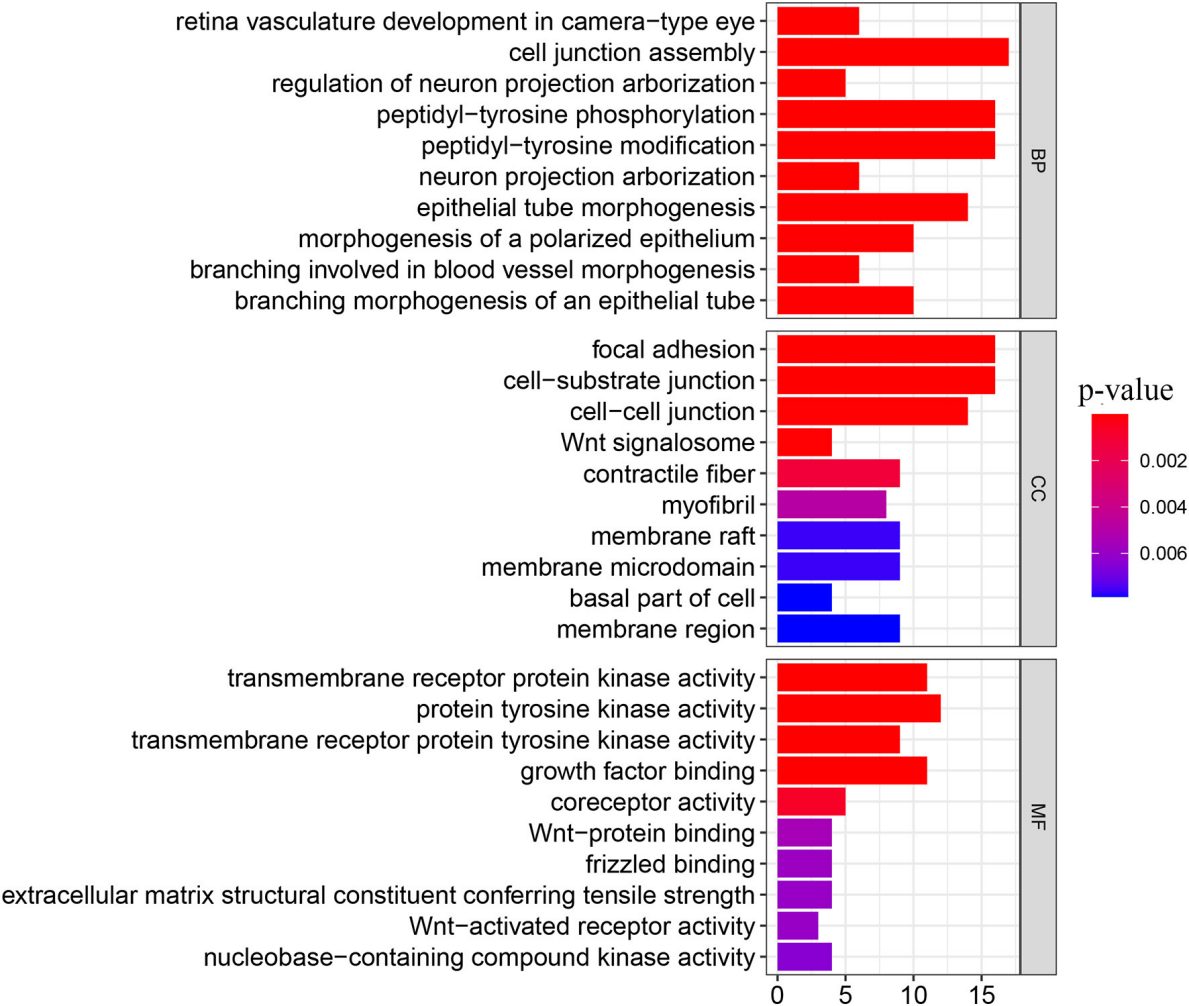
GO enrichment analysis. The y-axis shows top 10 significantly enriched BP, CC, MF categories, and the x-axis displays the enrichment scores of these terms (*P*-value < 0.05).

We obtained 51 FAT4-binding proteins by using the online tool STRING, and identified the top 100 FAT4-related targeted genes by using the GEPIA2 tool. We found that the expression of FAT4 was positively correlated with DCHS1 (R = 0.56), TTC28 (R = 0.53), PRKG1 (R = 0.53), TNS1 (R = 0.52), and TGFBR2 (R = 0.52) genes *via* the GEPIA2 tool. Additionally, we found that the top 5 FAT4-related targeted genes were positively correlated with FAT4 in the vast majority of cancers. Furthermore, the expression of top 5 genes (DCHS1, PRKG1, TGFBR2, TNS1, and TTC28) in the majority of detailed cancer types was shown in the form of heatmap (Figure 7). Moreover, association between FAT4 expression and genes involved in biological pathways was analyzed by GSEA. GSEA of BLCA indicated that low FAT4 expression groups were mainly enriched in calcium signaling pathway and chemokine signaling pathway. GSEA analysis of KIRC suggested low FAT4 expression groups were mainly involved in olfactory transduction and oxidative phosphorylation (Figure 8A). To further elucidate the specific mechanism of FAT4 gene in tumorigenesis, we performed KEGG analysis and GO enrichment analysis on FAT4-binding proteins and FAT4-related targeted genes. The corresponding barplot of KEGG indicated that the role of FAT4 in the pathogenesis of cancer may be related to human papillomavirus infection, Hippo signaling pathway, PI3K–Akt signaling pathway (Figure 8B). GO enrichment analysis included Biological process (BP), Cellular Component (CC) and Molecular Function (MF). GO enrichment analysis further indicated that most of these genes were related to the pathways or cell biology, such as peptidyl–tyrosine phosphorylation, cell junction assembly, protein tyrosine kinase activity, and others (Figure 9).

## Discussion

Through a literature search, we currently did not find any publications on a pan-cancer analysis of FAT4. FAT4 Potential functions and mechanisms in tumor progression and tumor immunology remained unclear. According to the data from the TCGA platform, we found the downregulation of FAT4 expression in 14 types of cancer, including BLCA, BRCA, COAD, KICH, KIRP, LIHC, LUAD, LUSC, PRAD, READ, THCA, UCEC, CESC, and GBM. Through Gene Expression Analysis, we speculated that FAT4 was a tumor suppressor gene. Studies suggested FAT4 expression was reduced in gastric adenocarcinoma and hepatocellular carcinoma tumor tissues, and FAT4 downregulation was associated with tumor grade (11, 12). Functional analysis using RNAi-mediated knockdown of FAT4 revealed an increased cancer cell growth and proliferation, suggesting the putative tumor suppressor role of FAT4 in hepatocellular carcinoma cancer (12). Studies have also shown that the tumor-suppressive functions of FAT4 were also identified in breast and gastric cancers (13, 14). These results were consistent with the findings of our current study. In addition, a prevalence screening confirmed mutations in FAT4 in 5% of gastric cancers, but the specific mechanism was still unclear (14).

By analyzing the relationship between the expression level of FAT4 in tumor tissues and tumor stage, we found with the increase of AJCC stage of KIRC tumor, the down-regulation of FAT4 increased, it suggesting a negative correlation between FAT4 and AJCC stage. In BRCA tissues, the expression level of Stage IV was the lowest, and the expression levels of Stage I and Stage III were relatively higher. In BLCA tissues, the expression level of Stage II was the lowest, and the expression level of Stage I was relatively higher. Referring to the low expression of FAT4 in KIRC, BLCA, and BRCA tissues, we speculated that the further low expression of FAT4 could promote the further malignant development of these tumors. In addition, the related study (15) has explored the mechanism of FAT4 promoting the further malignant development of Triple negative breast cancer (TNBC) cells, it has shown that the TNBC cell lines have enhanced proliferation ability, weakened apoptosis ability and enhanced invasion and migration ability after the reduction of FAT4 expression level. Low expression of FAT4 can promote the further malignant development of TNBC, which is consistent with the speculation in this manuscript. To the best of our knowledge, there were no specific reports on the tumor suppressive effect of FAT4 in KIRC and BLCA tissues, and further experiments will be required to determine whether this is indeed the case.

The survival analysis results suggested the expression level of FAT4 in different tumor types is of great significance to the survival prognosis of cancer patients. It was important to note that different conclusions are drawn for different cancer types. Our study suggested that patients with low FAT4 expression have a poor prognosis for HNSC, KIRC, and LIHC patients. The findings were consistent with the results of a previous study (16) that indicated that FAT4 deficiency may lead to uncontrolled tumor progression and unfavorable clinical outcome in hepatocellular carcinoma. We also found that BLCA patients with lower expression of FAT4 had a better prognosis.

Cancer-associated fibroblasts (CAFs) are a large heterogeneous mesenchymal cell population and an important component of the microenvironment in solid tumors. CAFs were shown to enhance tumor growth through secretion of growth factors, induction of angiogenesis, etc., (17). We calculated the correlation between immune infiltration and FAT4 expression levels in various cancer types. We found that there exists a statistically positive correlation between FAT4 expression and CAFs for most cancer types, such as ACC, BLCA, and BRCA based on EPIC, MCPCOUNTER, xCell, and TIDE algorithms. This finding possibly indicated that the correlation between FAT4 and CAFs infiltration was cancer-dependent, and with the deepening of CAFs-mediated infiltration, tumor cells may hijack FAT4 to protect tumor cells and promote tumor growth.

Correlation gene screening showed that FAT4 was significantly positively correlated with DCHS1, TTC28, PRKG1, TNS1, and TGFBR2 expression in tumors. PRKG1 was a cyclic cGMP-dependent protein kinase (PKG). There was a study provided that the interaction between Src and the NO/cGMP/PKG signaling pathway that was important for promoting DNA synthesis and cell proliferation in human ovarian cancer cells. The cGMP/PKG signaling pathway played an important role in the anti-apoptotic mechanism of ovarian cancer cell lines (18). The NO/cGMP/PKG signaling pathway has also been reported to protect human ovarian cancer cells against both spontaneous and cisplatin-induced apoptosis (19). TNS1 was a direct target of miR-31-5p, the tumor-promoting effects of miR-31-5p on LUAD cell functions are attenuated by TNS1 overexpression to some extent (20). TGFBR2(The type II transforming growth factor β receptor gene) was frequently frameshift mutated in several cancer types, especially in colorectal, endometrium, and gastric cancer cells (21).

GSEA of BLCA indicated that low FAT4 expression groups were mainly enriched in calcium signaling pathway and chemokine signaling pathway. Previous studies (22, 23) suggested that calcium signaling pathway and chemokine signaling pathway were associated with bladder cancer progression at both the Ta-T1 and T1-T2 stages. Few previous studies were found between FAT4 and KIRC.

KEGG pathway analysis indicated that the role of FAT4 in the pathogenesis of cancer may be related to human papillomavirus (HPV) infection, Hippo signaling pathway, and PI3K–Akt signaling pathway. HPV infection is a multi-step process that implies complex interactions of the viral particles with cellular proteins (24). Persistent HPV infection can eventually lead to clinical problems, varying from verrucous lesions to malignancies like cervical cancer, oral cancer, anus cancer, and so on (25). Hippo signaling pathway is an emerging signaling pathway that plays an important role in organ size control, tumorigenesis, metastasis, stress response, apoptosis, stem cell differentiation, and renewal during development and tissue homeostasis (26). Its dysregulation is one of the important regulatory factors of various cancers, such as liver cancer (27) and colorectal cancer (28). Functionally, FAT4 is known as an upstream member involved in the regulation of Hippo signaling pathway by activating YAP which is one of the main downstream effectors of the Hippo signaling pathway (29, 30). Additionally, there has been a study indicating that FAT4 inactivation promoted migration and invasion of endometrial cancer cells *via* inhibiting the Hippo pathway (31). The phosphoinositide 3-kinase (PI3K)–AKT pathway is the most commonly activated pathway in human cancers (32). FAT4 can regulate the activity of PI3K to promote autophagy and inhibit the EMT in part through the PI3K/AKT/mTOR and PI3K/AKT/GSK-3β signaling pathways (33). miR-107 promotes the growth and metastasis of gastric cancer via activation of PI3K-AKT signaling pathway by targeting FAT4, which may be a target for gastric cancer treatment (34). According to another recent study, Brucea javanica oil can inhibit the proliferation of hepatocellular carcinoma cells and induce apoptosis *via* the PI3K/AKT pathway (35).

In summary, FAT4 is abnormally downregulated in 14 types of cancer, including BLCA, BRCA, COAD, KICH, KIRP, LIHC, LUAD, LUSC, PRAD, READ, THCA, UCEC, CESC, and GBM tumor tissues, and its anomalous expression is related to the prognosis of tumors. This pan-cancer analysis showed that FAT4 expression was statistically correlated with clinical stages, clinical prognosis, immune infiltration, and FAT4-related gene enrichment analysis, which aided in understanding the role of FAT4 in tumorigenesis from the perspective of clinical tumor samples. Therefore, FAT4 can be used as a potential prognostic biomarker for various cancers prognosis.

## Data availability statement

The original contributions presented in the study are included in the article/supplementary material, further inquiries can be directed to the corresponding authors.

## Author contributions

Conception and design: WM, KM, and JW. Acquisition of data: JZ, KZ, and ZF. Processing of data: WM and JH. Drafting of the manuscript: WM. Critical revision of the manuscript for important intellectual content: JW and KM. The authors read and approved the final manuscript.

## Funding

This research was financially supported by Zhejiang Provincial Natural Science Foundation of China under Grant (No. LYQ20H300001).

## Conflict of interest

The authors declare that the research was conducted in the absence of any commercial or financial relationships that could be construed as a potential conflict of interest. The reviewer WL declared a shared affiliation with the author KZ to the handling editor at time of review.

## Publisher's note

All claims expressed in this article are solely those of the authors and do not necessarily represent those of their affiliated organizations, or those of the publisher, the editors and the reviewers. Any product that may be evaluated in this article, or claim that may be made by its manufacturer, is not guaranteed or endorsed by the publisher.

## Abbreviations

TCGA: The Cancer Genome Atlas
TMB: Tumor Mutational Burden
MSI: Microsatellite Instabilit
GSEA: Gene Set Enrichment analysis
KEGG: Kyoto Encyclopedia of Genes and Genomes
GO: Gene Ontology
RNA seq: RNA sequencing
SPM: Somatic Point Mutation
OS: Overall Survival
DSS: Disease Specific Survival
DFS: Disease Free Survival
PFS: Progression Free Survival
HR: Hazard Ratio
CAFs: Cancer-Associated Fibroblasts
HPV: Human Papillomavirus
PI3K: Phosphoinositide 3-Kinase.
